# Variation in Genes that Regulate Blood Pressure Are Associated with Glomerular Filtration Rate in Chinese

**DOI:** 10.1371/journal.pone.0092468

**Published:** 2014-03-21

**Authors:** May E. Montasser, Lawrence C. Shimmin, Dongfeng Gu, Jing Chen, Charles Gu, Tanika N. Kelly, Cashell E. Jaquish, Treva K. Rice, Dabeeru C. Rao, Jie Cao, Jichun Chen, De-Pei Liu, Paul K. Whelton, Lotuce Lee Hamm, Jiang He, James E. Hixson

**Affiliations:** 1 Human Genetics Center, School of Public Health, University of Texas Health Science Center at Houston, Houston, Texas, United States of America; 2 Cardiovascular Institute and Fu Wai Hospital, Chinese Academy of Medical Sciences, Beijing, China; 3 Tulane University School of Medicine, New Orleans, Louisiana, United States of America; 4 Washington University in School of Medicine, St. Louis, Missouri, United States of America; 5 Tulane University School of Public Health and Tropical Medicine, New Orleans, Louisiana, United States of America; 6 National Heart, Lung and Blood Institute, National Institute of Health, Bethesda, Maryland, United States of America; 7 National Laboratory of Medical Molecular Biology, Institute of Basic Medical Sciences, Chinese Academy of Medical Sciences, Beijing, China; University of São Paulo School of Medicine, Brazil

## Abstract

Chronic kidney disease (CKD) can be a consequence of diabetes, hypertension, immunologic disorders, and other exposures, as well as genetic factors that are still largely unknown. Glomerular filtration rate (GFR), which is widely used to measure kidney function, has a heritability ranging from 25% to 75%, but only 1.5% of this heritability is explained by genetic loci that have been identified to date. In this study we tested for associations between GFR and 234 SNPs in 26 genes from pathways of blood pressure regulation in 3,025 rural Chinese participants of the “Genetic Epidemiology Network of Salt Sensitivity” (GenSalt) study. We estimated GFR (eGFR) using baseline serum creatinine measurements obtained prior to dietary intervention. We identified significant associations between eGFR and 12 SNPs in 6 genes (*ACE, ADD1, AGT, GRK4*, *HSD11B1*, and *SCNN1G*). The cumulative effect of the protective alleles was an increase in mean eGFR of 4 mL/min per 1.73 m^2^, while the cumulative effect of the risk alleles was a decrease in mean eGFR of 3 mL/min per 1.73 m^2^. In addition, we identified a significant interaction between SNPs in *CYP11B1* and *ADRB2*. We have identified common variants in genes from pathways that regulate blood pressure and influence kidney function as measured by eGFR, providing new insights into the genetic determinants of kidney function. Complex genetic effects on kidney function likely involve interactions among genes as we observed for *CYP11B1* and *ADRB2*.

## Introduction

Chronic kidney disease (CKD) is a major risk factor for cardiovascular disease and all-cause mortality, placing a huge burden on the health care system [1], [2]. CKD is a complex trait, regulated by interactions of several environmental and genetic factors [3]. CKD can arise as a consequence of diabetes, hypertension, immunologic disorders (such as lupus or primary glomerulonephritis), and a variety of other exposures. While the genetic causes of monogenic forms of renal diseases are well established, those contributing to the common forms of CKD are still largely unknown [4]. Recent meta analyses of genome wide association studies (GWAS) identified several loci for kidney function indices and CKD, collectively explaining a very small fraction of the variability of these traits, leaving most of their genetic components undetermined [5]. Earlier linkage analyses and candidate gene studies also produced inconsistent or unconfirmed results [6]–[9]. Most study populations were ascertained on the basis of disease status, resulting in enrichment for diabetes, obesity, and hypertension, thus complicating genetic studies of CKD [10], [11].

Genetic studies of CKD can benefit from a pathway-based targeted approach that includes testing for genetics and environmental interactions [12] for an intermediate quantitative trait [13] such as glomerular filtration rate (GFR) that is widely used to measure kidney function. GFR is a complex trait with an estimated heritability of 25–75% [14]. However, only about 1.5% of its variability has been explained by the genetic loci that have been identified so far [5].

In the current study, we examined the genetic factors that may influence GFR in rural Chinese participants of the “Genetic Epidemiology Network of Salt Sensitivity” (GenSalt) study, who did not have clinical evidence of overt CKD. Estimated GFR (eGFR) values were calculated from serum creatinine measurements obtained during a three-day observation period preceding dietary intervention, while the participants consumed their usual diet. We tested single nucleotide polymorphisms (SNPs) in 26 genes from pathways of blood pressure regulation for association with eGFR measures. This genetic study of GFR provides insight into the genetic determinants of kidney function in a general population sample of individuals without CKD, and potentially into the initiation and progression of CKD.

## Materials and Methods

### Ethics statement

Institutional review boards at Tulane University Health Sciences Center, Washington University School of Medicine, University of Texas School of Public Health, Fu Wai Hospital and Chinese National Human Genome Center at Beijing, Chinese Academy of Medical Sciences approved the GenSalt study. Written informed consents for the baseline observation and for the intervention program were obtained from each participant.

### Study population

The GenSalt Study was conducted in Han Chinese families living in six rural villages in Northern China. Families were recruited through 18–60 year old probands who were either prehypertensive or had stage-1 hypertension (SBP 130–160 mm Hg and/or DBP 85–100 mm Hg), but had never been treated with antihypertensive medication. Parents, spouses, siblings, and offspring were invited to participate in the study. Family members were excluded if they had stage-2 hypertension, a history of CVD, diabetes, or heavy alcohol consumption, or were pregnant, on a low sodium diet or taking anti-hypertensive medications. A total of 3,025 individuals in 631 families participated in this study. A large number of demographic, anthropomorphic, and medical variables were measured in GenSalt participants. More information regarding participants recruitment and measurements are available elsewhere [15]. Institutional Review Board approval for this study was obtained at all of the participating institutions and all study participants signed an informed consent document.

### Phenotype measurements

Serum creatinine was measured during a 3-day baseline observation period while the study participants consumed their usual diet prior to a GenSalt dietary intervention. During this period, an overnight fasting blood sample was obtained from each participant by venipuncture. This was used to measure serum creatinine by the modified kinetic Jaffe reaction method. GFR was estimated using an amended formulation of the Modification of Diet in Renal Disease (MDRD) study equation, specifically designed for use in healthy individuals [16]: eGFR in mL/min per 1.73 m^2^  =  216 × (serum creatinine in mg/dL)^−0.490^ × (age in years)^−0.192^ × 0.923 (if female).

### Gene and SNP selection and genotyping

The GenSalt genotyping effort focused on 26 blood pressure candidate genes. The genes were selected based on their presumed role in blood pressure homeostasis and being a part of blood pressure regulation pathways such as the renin angiotensinsystem (*REN*, *RENBP*, *AGT*, *AT2R1*, *AT2R2*, *ACE*), the aldosterone system (*CYP11B1*, *CYP11B2*, *MLR*, *HSD11B1*, *HSD11B2*, *CYP3A5*), the endothelial system (*EDN1*, *NOS3*, *SELE*), the sympathetic nervous system (*GRK4*, *ADRB2*), alternative renin angiotensinsystem pathway (*ACE2*, *APLN*, *AGTRL1*), as well as atrial natriuretic peptide genes (*NPR3*, *NPPA*), sodium channels genes (*SCNN1B*, *SCNN1G*), and intracellular messengers genes (*GNB3*, *ADD1*). We selected 234 SNPs within these genes based on linkage disequilibrium (LD) structure in the Chinese population from the International HapMap project [17]. SNP genotyping was performed using the SNPlex platform (Applied Biosystems, Foster City CA) according to the manufacturer's protocol [18]. We excluded 41 SNPs in three genes from the association analysis due to low call rate (<80%), low minor allele frequency (MAF <0.05), or severe deviation from Hardy-Weinberg Equilibrium (HWE) (p<0.001). Detailed information concerning the remaining 193 SNPs within 24 genes is listed in **Table S1**.

### Statistical analysis

Plink and PedCheck programs were used to assess the Mendelian consistency of SNP genotype data [19], [20]. Programs from the Affected-Sib-Pair Interval Mapping and Exclusion package (ASPEX) and the Graphical Representation of Relationships (GRR) package were used to check for potential misreported relationships within pedigrees [21], [22]. Haploview (Broad Institute, Boston MA) was used for SNP descriptive statistics [23]. The Generalized Estimation Equation (GEE) method was used to test for associations between eGFR and the genetic variants, accounting for familial correlation [24]. Values for eGFR were adjusted for significant covariates including age, age^2^, age^3^, gender, BMI, high density lipoprotein cholesterol (HDL-C), hypertension, and field center. Neither smoking nor alcohol consumption was significantly associated with eGFR and were not included as covariates in statistical analysis. GEE analysis was performed with SAS 9.1 using proc genmod, and exchangeable working correlation matrix. False Discovery Rate (FDR) was used to correct for the multiple testing in GEE analysis [25]. For interactions among genes, we used the Generalized Multifactor Dimensionality Reduction program (GMDR) [26] to determine joint effects of each pair of SNPs. The best model identified by GMDR for eGFR was verified using GEE to account for familial correlation. Several web algorithms and data bases were used for SNP annotation and bioinformatics analysis including UCSC [27], SNPnexus [28], [29], PolyPhen-2 [30], SIFT [31], and FastSNP [32].

## Results

**Table 1** presents the basic characteristics of the 3,025 GenSalt participants who were included in this study. The study sample consisted of healthy free living members of three-generation families with an average age of 50 years, and approximately equal proportions of males and females. Their average values for BMI, HDL-C, serum creatinine, and eGFR were all within normal ranges. **Table S1** shows characteristics of the 193 SNPs within 26 loci involved in blood pressure regulation that were chosen according to LD in the Han Chinese population (International HapMap project).

**Table 1:** Basic characteristics of the study subjects.

	Healthy subjects
**N**	3025
**Age**	50.0± 16.6
**Male %**	51.3
**Hypertension %**	17
**Smokers %**	34.5
**Alcohol consumers %**	27
**Body Mass Index**	23.1 ± 3.2
**HDL (mg/dL)**	51.47 ± 11.3
**Serum creatinine (mg/dL)**	0.93 ± 0.21
**eGFR (mL/min per 1.73 m^2^)**	104.91 ± 14.07

HDL: High Density Lipoprotein, eGFR: estimated Glomerular Filtration Rate.

Values are mean ± standard deviation for Age, Body Mass Index, HDL, serum creatinine, and eGFR.

**Figure 1:**
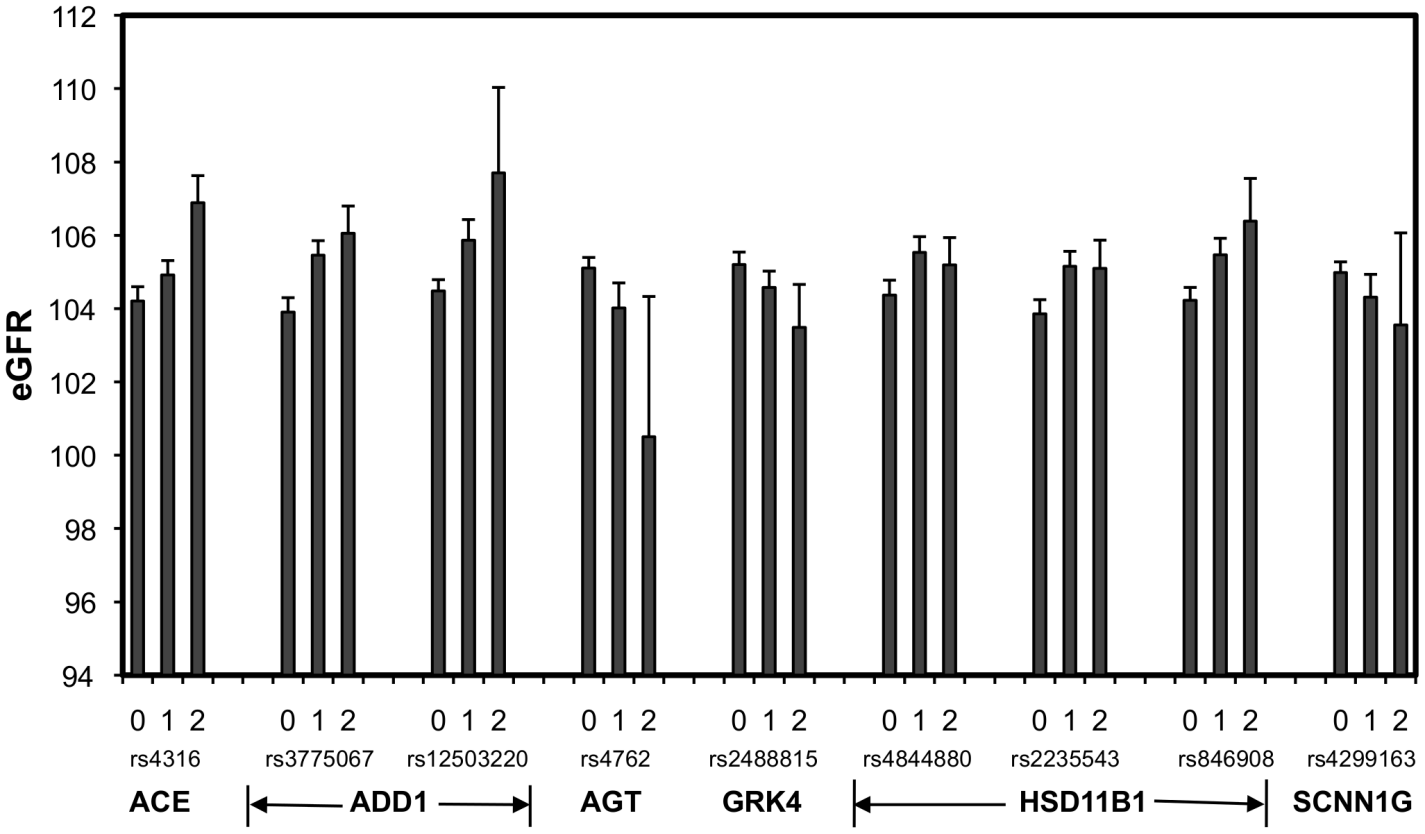
Mean eGFR values and standard errors for genotypes of SNPs that showed significant associations (0, homozygotes for common alleles; 1, heterozygotes; 2, homozygotes for minor alleles).

**Table 2** presents results of genetic analysis that identified 12 SNPs in six genes that showed significant associations with eGFR (p<0.05). The four significant SNPs in *ACE* were highly correlated with r^2^ ranging from 0.78 to 0.96. The three SNPs in the hydroxysteroid 11-beta dehydrogenase 1 gene (*HSD11B1*) were less correlated (r^2^ from 0.35 to 0.43), and the two significant SNPs in the alpha-adducin gene (*ADD1*) were not significantly correlated (r^2^  = 0.17). The FDR for all significant p-values was 0.59, which means that seven of these 12 significant SNPs might have been false positives. **Figure 1** shows the average eGFR values for each of the three genotypes for nine of the significant SNPs (excluding three highly correlated *ACE* SNPs). Three of the significant SNPs (AGT_rs4762, GRK4_rs2488815, and SCNN1G_rs4299163) can be considered as risk SNPs where the additive effect of the minor allele was associated with lower values of eGFR. The other significant SNPs can be considered as protective, where the minor allele was associated with higher eGFR values.

**Table 2:** SNPs that showed significant associations with eGFR in GenSalt participants.

Gene	Chr.	SNP	Region	HWpval	Call Rate	MAF	Maj/Min	P*
ACE	17	rs4316	exon	0.0917	97.2	0.353	T/C	0.0077
ACE	17	rs4343	exon	0.4012	92.7	0.354	A/G	0.0170
ACE	17	rs4353	intron	0.2622	97.6	0.393	G/A	0.0181
ACE	17	rs4331	exon	0.2927	96.2	0.353	G/A	0.0313
ADD1	4	rs3775067	intron	0.934	97.1	0.34	C/T	0.0061
ADD1	4	rs12503220	utr	0.3043	93.1	0.134	G/A	0.0231
AGT	1	rs4762	exon	0.3746	95.6	0.073	C/T	0.0051
GRK4	4	rs2488815	intron	0.0255	96.7	0.206	C/T	0.0279
HSD11B1	1	rs4844880	utr	0.6649	89.5	0.356	T/A	0.0166
HSD11B1	1	rs2235543	utr	0.0703	92.5	0.35	C/T	0.0308
HSD11B1	1	rs846908	intergenic	0.9629	92.5	0.251	G/A	0.0419
SCNN1G	16	rs4299163	intron	0.8433	96.6	0.103	G/C	0.0247

HWpval: Hardy-Weinberg p value, MAF: minor allele frequency, Maj/Min: Major/Minor allele.

*p values adjusted for age, age^2^, age^3^, gender, BMI, high density lipoprotein cholesterol (HDL-C), hypertension, field center, and family structure.

**Figure 2:**
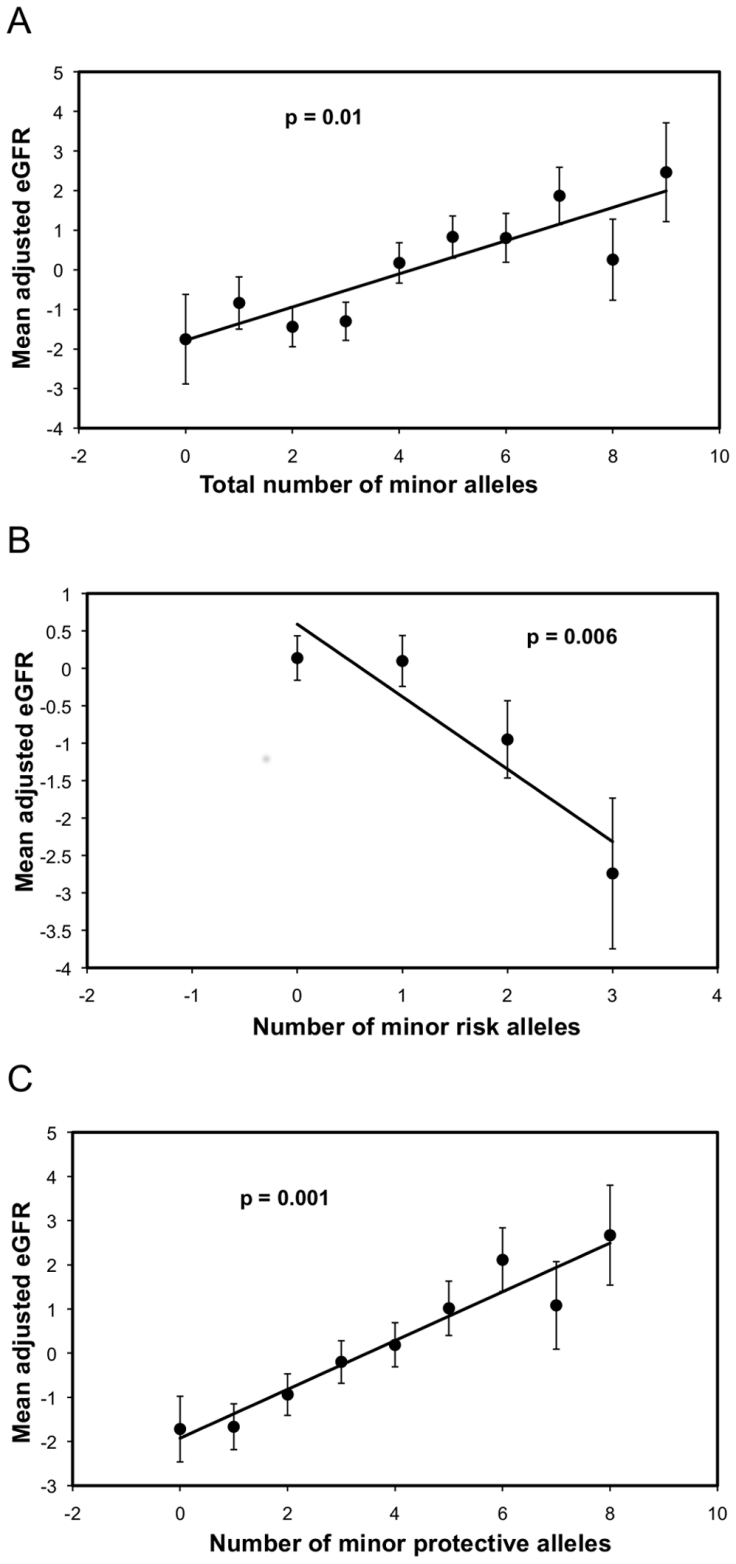
The cumulative effect of the minor alleles in all of the 9 significant SNPs (Panel A), the 6 protective SNPs (Panel B), and the 3 risk SNPs (Panel C) on the value of the mean adjusted eGFR. The best fitting trend line and p value from t-tests between those with no minor allele and those with the largest possible number of minor alleles in each category are shown.

**Figure 2** shows the cumulative effects on mean adjusted eGFR values for carriers of the minor alleles for all nine significant SNPs (**Panel A**) and separately for the six protective alleles (**Panel B**) and three risk alleles (**Panel C**). Carriers with increasing numbers of the minor protective alleles had up to more than 4 mL/min per 1.73 m^2^higher mean eGFR values (p = 0.001) (**Figure 2** **, Panel B**). Carriers with increasing numbers of the minor risk alleles had mean eGFR values that were as much as almost 3 mL/min per 1.73 m^2^ lower (p = 0.006) **(** **Figure 2** **, Panel C)**.

**Figure 3:**
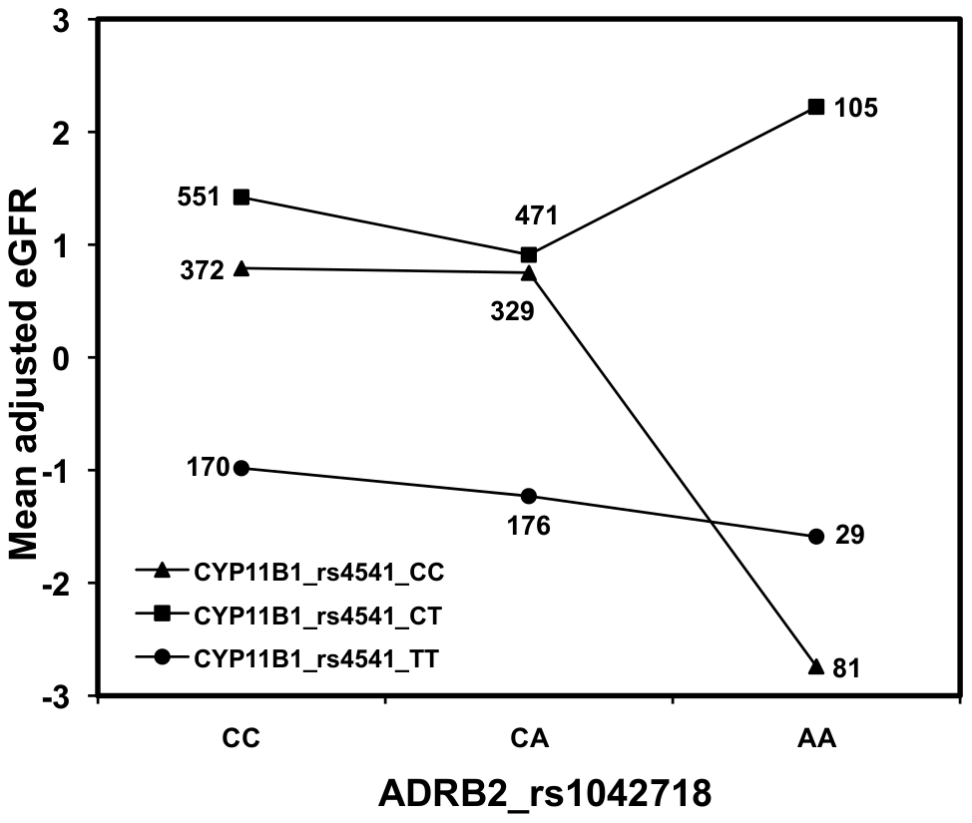
Mean adjusted eGFR as a result of the genotypic interaction between CYP11B1_rs4541 and ADRB2_rs1042718. The data points represent the eGFR for the nine possible combinations of the three *ADRB2* genotypes versus each of the three possible *CYP11B1* genotypes. The number of individuals at each point is provided.

We also tested the SNPs for effects of gene by gene interactions (GxG) on eGFR. We identified a joint effect on eGFR between a nonsynonymous SNP in the gene for cytochrome P450, family 11, subfamily B, polypeptide 1 (CYP11B1_rs4541, Ala386Val) and a synonymous SNP in the beta-2-adrenergic receptor gene (ADRB2_rs1042718). **Figure 3** shows the joint effects on mean eGFR values of interactions between CYP11B1_rs4541 and ADRB2_rs1042718 genotypes. The mean adjusted eGFR value in homozygotes for the ADRB2_rs1042718 minor allele (AA) depended on their genotype for CYP11B1_rs4541. Homozygotes for the minor allele of ADRB2_rs1042718 (AA) who are also homozygous for the major allele of CYP11B1_rs4541 (CC) had the lowest mean eGFR values. Homozygotes for the minor allele of ADRB2_rs1042718 (AA) who are heterozygous for CYP11B1_rs4541 (CT) had the highest eGFR. The difference between these two joint genotypes was approximately 5 mL/min per 1.73 m^2^.

## Discussion

The overall goal of this study was to conduct a comprehensive examination of the effects of variability in genes from pathways of blood pressure regulation on renal GFR. The GenSalt study cohort was comprised of rural Han Chinese villagers to minimize the genetic heterogeneity that is encountered in most association studies that are conducted in admixed urban populations. None of our study participants were taking antihypertensive medication, so the complexity associated with the antihypertensive drugs is absent from our study. In addition, we employed an amended version of the MDRD eGFR equation that was specifically designed for use in healthy free living individuals and eliminated the underestimation of GFR with the equation that was previously in use [16].

In our analyses of individual SNPs, the Thr207Met polymorphism (rs4762) in the angiotensinogen gene (*AGT*) showed the strongest association with eGFR (**Table 2**). *AGT* plays a role in the renin-angiotensin system (RAS), a primary pathway in blood pressure regulation with strong influences on cardiovascular and renal disease. *AGT* encodes preangiotensinogen in the liver, which is subsequently cleaved by renin to generate angiotensin I. Angiotensin I converting enzyme (*ACE*), converts angiontensin I to angiotensin II [33], [34], a potent vasoconstrictor that also affects renal hemodynamics by decreasing renal cortical blood flow, total renal plasma flow, urinary sodium excretion, and GFR [33], [34]. Moreover, angiotensin II increases glomerular capillary pressure, potentially contributing to glomerulosclerosis [35], [36]. AGT_rs4762 (Thr207Met) is a probably damaging SNP [30], [31] as it substitutes a non-polar amino acid (methionine) for a polar amino acid (threonine). Furthermore, threonine at this position in *AGT* is highly conserved among divergent species ranging from human to zebrafish [37]. Previous studies in Asians have identified associations of AGT_rs4762 with diabetic nephropathy in Taiwanese patients [38] and hypertension in different Asian populations based on meta-analysis [37].

We also found significant associations with eGFR for four correlated SNPs in the *ACE* gene, another key player in the RAS pathway of blood pressure regulation. Three of these SNPs (ACE_rs4316, ACE_rs4331, ACE_rs4343) are exonic variants, but do not cause amino acid substitutions (synonymous SNPs). ACE_rs4343, was previously reported to be significantly associated with diabetic nephropathy in an Asian Indian population [39].

Our analysis identified a significant association of eGFR with two intronic SNPs (rs3775067 and rs12503220) in the adducin 1 gene (*ADD1*). *ADD1* encodes the alpha subunit of the cytoskeleton protein adducin, which plays an important role in hypertension and renal function via sodium homeostasis [40]. Many previous studies have reported associations of *ADD1* variants with hypertension, renal functions and renal diseases, in different populations including Chinese [40]–[48].

Another SNP that showed associations in our study was rs2488815, an intronic SNP in the G protein_coupled receptor kinase 4 (*GRK4*) gene, a major player in sodium homeostasis and blood pressure regulation [49]. *GRK4* is expressed in the renal proximal tubule, where about 70% of renal sodium reabsorption takes place. Increased *GRK4* activity leads to decreased dopamine signaling and increased AngII receptor expression and function, both of which increase sodium retention and blood volume which ultimately leads to hypertension [50], [51]. *GRK4* variants have been shown to be associated with hypertension and blood pressure traits in different populations including Han Chinese [52].

Three SNPs in the hydroxysteroid 11beta dehydrogenase1 gene (*HSD11B1*) were associated with eGFR in the current study. *HSD11B1* is a NADP dependent enzyme that functions in the proximal tubule and medullary interstitial cells of the human kidney. *HSD11B1* plays a role in the metabolism of the endogenous glucocorticoids, which in turn modulate sodium homeostasis, renal blood flow, and GFR [53]. *HSD11B1* enzymatic activities are thought to be involved in obesity, hypertension, and other components of the metabolic syndrome. *HSD11B1* overexpression in mice has been associated with dose-dependent hypertension and *AGT* expression in liver [54]–[56].

Our analyses of individual SNPs identified associations of eGFR with an intronic SNP (rs4299163) in the gene encoding the gamma subunit of the epithelial sodium channel gene (*SCNN1G*). Epithelial sodium channels (*ENaC*), are the main regulators for sodium transport in the kidney [57], [58], and rare variants in *SCNN1G* cause Liddle Syndrome, a monogenic form of hypertension [59]. Other variants in *SCNN1G* cause pseudohypoaldosteronism type 1, a rare inherited form of renal tubular acidosis [60], [61]. Many studies have identified linkage of SBP with the region that contains *SCNN1G* on chromosome 16 [62]. A fine mapping study of this region detected associations of SBP with three *SCNN1G* intronic SNPs, including rs4299163 [62].

In addition to analyses of individual SNPs, we tested for interactions among genes (GxG interactions) that influence eGFR. Our GxG analyses identified a joint effect on eGFR between a conservative nonsynonymous SNP rs4541 (Ala386Val) in *CYP11B1* and a synonymous SNP rs1042718 in *ADRB2*. *CYP11B1* is one of the cytochrome p450 genes encoding 11β hydroxylase, a protein involved in the synthesis of cortisol in the adrenal cortex [63]. Cortisol is associated with Cushing's syndrome, hypertension of chronic renal failure, hypertension related to low birth weight, and essential hypertension [64], [65]. Glucocorticoid-Remediable Aldosteronism, a rare form of hypertension, is caused by a gene fusion between *CYP11B1* and *CYP11B2* [66]. *ADRB2* encodes the beta 2 adrenergic receptor, a member of the G-protein superfamily receptors, playing a role in metabolism regulation and also in blood pressure regulation by mediating vasodilation and vascular resistance [67], [68]. *ADRB2* SNPs were previously found to be associated with hypertension and blood pressure traits in different populations including Han Chinese but many of the results are inconsistent [68]–[70]. ADRB2_rs1042718, the SNP showing significant GxG interaction, is part of a haplotype recently found to be associated with weight, insulin, and homeostasis model assessment (HOMA) score in Korean adolescents [71]. The exact mechanism of interaction between these two coding region SNPs in relation to eGFR warrants further investigation.

We compared the results of our association study with a recently published meta-analysis of kidney function traits in several East Asian populations, including GenSalt [72]. The SNPs with significant associations in our study (Table 2) were not significantly associated with eGFR in the meta-analysis. This discrepancy could stem from differences in the study populations since we included only Han Chinese from Northern China, while the meta-analysis included Japanese, Malay, Indian, Korean, and Chinese. GenSalt participants were relatively healthy free living individuals from three-generation families, while the meta-analysis included hospital and population based cohorts with no exclusion of diseased individuals. In addition, we employed an equation to calculate eGFR that was specifically designed for use in healthy individuals [16], while the meta-analysis used a different equation specific for Japanese individuals [73].

The genes included in this study were all in pathways known to be involved in regulation of blood pressure which may play an important role in regulating kidney function. However, there are established overlaps, such as the RAS pathway which is involved in renal function decline since treatment of patients with renin-angiotensin inhibitors slows the progression of kidney disease [74]–[76]. Such protective effect of RAS blocking on kidney function may be through both blood pressure and non-blood pressure dependent mechanisms. Our study was not designed to determine whether these genes impact GFR independent of their effects on blood pressure, or whether their effects on GFR are linked to the same pathophysiologic cascades as their involvement in hypertension. However, their significant association with kidney function remains after adjustment for hypertension.

In conclusion, we have identified common variants in genes from pathways of blood pressure regulation and their interactions that influence kidney function, providing new insights into the genetic determinants of kidney function. A longitudinal association between these common variants and changes in kidney function remains to be investigated.

## Supporting Information

Table S1 **SNP characteristics.** Detailed information about the 193 SNPs used in analysis.
(DOC)
