# Supplementary material for: Variation in Genes that Regulate Blood Pressure Are Associated with Glomerular Filtration Rate in Chinese
Source: PLoS One. 2014 Mar 21;9(3):e92468. doi: 10.1371/journal.pone.0092468 (PMC3962404; doi:10.1371/journal.pone.0092468)
Supplement: Table S1 — SNP characteristics. Detailed information about the 193 SNPs used in analysis. (DOC) [file pone.0092468.s001.doc]

Table S1. SNP characteristics

| **Gene** | **SNP** | **Region** | **HWpval** | **MAF** | **Maj/Min** |
| --- | --- | --- | --- | --- | --- |
| ACE | rs12451328 | utr | 0.1171 | 0.2 | C/A |
| ACE | rs4316 | exon | 0.0917 | 0.353 | T/C |
| ACE | rs4331 | exon | 0.2927 | 0.353 | G/A |
| ACE | rs4343 | exon | 0.4012 | 0.354 | A/G |
| ACE | rs4353 | intron | 0.2622 | 0.393 | G/A |
| ACE | rs4362 | exon | 0.5605 | 0.395 | C/T |
| ACE | rs4459610 | exon | 0.8343 | 0.246 | A/T |
| ACE | rs4461142 | intron | 0.8179 | 0.426 | C/T |
| ACE | rs4968591 | utr | 0.8236 | 0.205 | T/C |
| ACE | rs8066276 | intron | 0.5546 | 0.249 | T/C |
| ADD1 | rs12503220 | utr | 0.3043 | 0.134 | G/A |
| ADD1 | rs1263359 | utr | 0.8974 | 0.431 | T/C |
| ADD1 | rs3775067 | intron | 0.934 | 0.34 | C/T |
| ADD1 | rs4690002 | intergenic | 0.6737 | 0.43 | C/T |
| ADD1 | rs4961 | exon | 0.5563 | 0.471 | T/G |
| ADD1 | rs4963 | exon | 0.8055 | 0.471 | G/C |
| ADRB2 | rs1042713 | exon | 0.4201 | 0.426 | A/G |
| ADRB2 | rs1042714 | exon | 0.5558 | 0.112 | C/G |
| ADRB2 | rs1042718 | exon | 0.8246 | 0.302 | C/A |
| ADRB2 | rs1042719 | exon | 1 | 0.436 | G/C |
| ADRB2 | rs11168070 | promoter | 0.6468 | 0.114 | C/G |
| ADRB2 | rs11959427 | promoter | 0.532 | 0.114 | T/C |
| ADRB2 | rs12654778 | promoter | 0.1714 | 0.383 | G/A |
| ADRB2 | rs1864931 | intergenic | 0.8973 | 0.185 | C/T |
| ADRB2 | rs3857420 | intergenic | 0.7987 | 0.482 | C/T |
| AGT | rs2006765 | intergenic | 0.0472 | 0.189 | C/T |
| AGT | rs2071404 | promoter | 0.5625 | 0.177 | G/T |
| AGT | rs2071405 | promoter | 0.3971 | 0.17 | G/A |
| AGT | rs2071406 | promoter | 0.1631 | 0.132 | T/C |
| AGT | rs2478545 | intron | 0.7146 | 0.258 | C/T |
| AGT | rs3789670 | intron | 0.7536 | 0.179 | C/T |
| AGT | rs3789671 | intron | 0.8913 | 0.453 | T/G |
| AGT | rs4762 | exon | 0.3746 | 0.073 | C/T |
| AGT | rs5046 | promoter | 0.6532 | 0.177 | C/T |
| AGT | rs5049 | promoter | 0.6333 | 0.18 | G/A |
| AGT | rs699 | exon | 0.2464 | 0.203 | C/T |
| AGT | rs7536290 | intergenic | 0.6594 | 0.214 | A/G |
| AGT | rs943580 | intergenic | 0.063 | 0.187 | G/A |
| AGTR1 | rs1492078 | promoter | 0.7428 | 0.2 | G/A |
| AGTR1 | rs5186 | utr | 0.3679 | 0.056 | A/C |
| AGTR1 | rs6801836 | utr | 0.0665 | 0.158 | T/C |
| AGTR1 | rs7427876 | intron | 0.1447 | 0.103 | G/A |
| AGTR1 | rs931490 | utr | 1 | 0.111 | A/G |

HWpval: Hardy-Weinberg p value, MAF: minor allele frequency,

Maj/Min: Major/Minor allele.

Table S1. SNP characteristics. Continued

| **Gene** | **SNP** | **Region** | **HWpval** | **MAF** | **Maj/Min** |
| --- | --- | --- | --- | --- | --- |
| AGTR1 | rs2131127 | utr | 0.5169 | 0.382 | C/T |
| AGTR1 | rs2638360 | utr | 1 | 0.108 | T/C |
| AGTR1 | rs275645 | intergenic | 0.9849 | 0.206 | A/G |
| AGTR1 | rs275646 | intergenic | 0.7715 | 0.097 | C/T |
| AGTR1 | rs275652 | promoter | 0.9142 | 0.149 | A/C |
| AGTR1 | rs3772616 | utr | 0.6027 | 0.17 | G/A |
| AGTR1 | rs389566 | utr | 0.1787 | 0.202 | T/A |
| AGTR1 | rs409742 | intergenic | 0.9636 | 0.15 | T/C |
| APLNR | rs2282623 | utr | 1 | 0.398 | C/T |
| APLNR | rs7119375 | intergenic | 0.374 | 0.231 | G/A |
| APLNR | rs721608 | intergenic | 0.6947 | 0.147 | A/G |
| BDKRB1 | rs2071084 | exon | 0.9699 | 0.233 | G/A |
| BDKRB2 | rs11847625 | utr | 0.8985 | 0.205 | G/C |
| BDKRB2 | rs2069575 | utr | 0.8574 | 0.199 | G/A |
| BDKRB2 | rs2069595 | intergenic | 0.9093 | 0.069 | C/T |
| BDKRB2 | rs2069600 | intergenic | 0.3274 | 0.183 | C/T |
| BDKRB2 | rs4905470 | utr | 0.4501 | 0.219 | G/A |
| BDKRB2 | rs4905474 | utr | 0.7708 | 0.383 | G/A |
| BDKRB2 | rs5224 | exon | 0.2305 | 0.176 | G/A |
| BDKRB2 | rs8016905 | utr | 0.1159 | 0.305 | G/A |
| BDKRB2 | rs945032 | promoter | 0.755 | 0.146 | G/A |
| BDKRB2 | rs945039 | utr | 0.4991 | 0.425 | C/T |
| CYP11B1 | rs4534 | exon | 0.2127 | 0.431 | G/A |
| CYP11B1 | rs4541 | exon | 1 | 0.398 | C/T |
| CYP11B1 | rs5283 | exon | 0.811 | 0.309 | C/T |
| CYP11B1 | rs6410 | exon | 0.1574 | 0.283 | G/A |
| CYP11B1 | rs7386926 | utr | 0.5771 | 0.159 | C/A |
| CYP11B1 | rs7463600 | intergenic | 0.4699 | 0.158 | C/A |
| CYP11B2 | rs1799998 | promoter | 0.7006 | 0.295 | T/C |
| CYP11B2 | rs4536 | exon | 0.7879 | 0.471 | G/A |
| CYP11B2 | rs7463212 | intergenic | 0.7961 | 0.318 | T/A |
| CYP3A5 | rs3800959 | intergenic | 0.563 | 0.156 | T/C |
| CYP3A5 | rs4646450 | intergenic | 0.3073 | 0.272 | C/T |
| CYP3A5 | rs776746 | intergenic | 0.1377 | 0.3 | G/A |
| EDN1 | rs1476046 | intron | 0.609 | 0.275 | G/A |
| EDN1 | rs1630736 | intron | 0.9849 | 0.444 | T/C |
| EDN1 | rs1800541 | intergenic | 0.9669 | 0.202 | T/G |
| EDN1 | rs1800543 | intron | 0.9566 | 0.275 | T/C |
| EDN1 | rs2859338 | intergenic | 0.6968 | 0.067 | G/A |
| EDN1 | rs3087459 | promoter | 0.4572 | 0.207 | A/C |
| EDN1 | rs5370 | exon | 0.8251 | 0.271 | G/T |

HWpval: Hardy-Weinberg p value, MAF: minor allele frequency,

Maj/Min: Major/Minor allele.

Table S1. SNP characteristics. Continued

| **Gene** | **SNP** | **Region** | **HWpval** | **MAF** | **Maj/Min** |
| --- | --- | --- | --- | --- | --- |
| GNB3 | rs2301339 | intron | 0.3346 | 0.453 | G/A |
| GNB3 | rs3213430 | promoter | 0.5615 | 0.317 | T/C |
| GNB3 | rs3213431 | promoter | 1 | 0.106 | T/C |
| GNB3 | rs4963516 | intergenic | 0.8347 | 0.329 | A/C |
| GNB3 | rs5446 | utr | 0.1297 | 0.201 | C/T |
| GRK4 | rs1024323 | exon | 0.4679 | 0.195 | G/A |
| GRK4 | rs1801058 | exon | 0.4891 | 0.43 | T/C |
| GRK4 | rs2471347 | intergenic | 0.0956 | 0.413 | C/T |
| GRK4 | rs2488813 | promoter | 0.6736 | 0.132 | T/A |
| GRK4 | rs2488815 | intron | 0.0255 | 0.206 | C/T |
| GRK4 | rs2857844 | intron | 0.3193 | 0.353 | T/C |
| GRK4 | rs2960306 | exon | 1 | 0.12 | G/T |
| HSD11B1 | rs11808690 | intron | 0.562 | 0.21 | T/G |
| HSD11B1 | rs12086634 | intron | 0.5238 | 0.224 | T/G |
| HSD11B1 | rs12091013 | intron | 0.7338 | 0.176 | G/T |
| HSD11B1 | rs2205985 | intergenic | 0.6856 | 0.396 | G/A |
| HSD11B1 | rs2235543 | utr | 0.0703 | 0.35 | C/T |
| HSD11B1 | rs2282739 | intron | 0.8942 | 0.487 | T/C |
| HSD11B1 | rs2282740 | intron | 0.2321 | 0.288 | C/A |
| HSD11B1 | rs3753519 | utr | 1 | 0.31 | G/A |
| HSD11B1 | rs4844488 | intron | 0.1458 | 0.263 | A/G |
| HSD11B1 | rs4844880 | utr | 0.6649 | 0.356 | T/A |
| HSD11B1 | rs846908 | intergenic | 0.9629 | 0.251 | G/A |
| KLK1 | rs2659058 | intron | 0.5914 | 0.21 | A/G |
| KLK1 | rs266112 | intron | 0.3503 | 0.099 | G/C |
| KLK1 | rs266116 | intergenic | 0.773 | 0.217 | T/A |
| KLK1 | rs3745522 | exon | 0.7412 | 0.496 | G/T |
| KLK1 | rs5517 | exon | 0.9406 | 0.409 | G/A |
| LNPEP | rs1423357 | intron | 0.0015 | 0.335 | T/A |
| LNPEP | rs2287902 | intron | 0.2465 | 0.074 | T/G |
| LNPEP | rs2303138 | exon | 0.1478 | 0.434 | G/A |
| LNPEP | rs3797787 | intron | 0.5849 | 0.054 | A/G |
| NOS3 | rs10277237 | intergenic | 0.6952 | 0.247 | A/G |
| NOS3 | rs1800781 | intron | 0.6814 | 0.084 | G/A |
| NOS3 | rs3918227 | intron | 0.8172 | 0.06 | C/A |
| NOS3 | rs743507 | intron | 0.7215 | 0.257 | A/G |
| NOS3 | rs7830 | intron | 0.0648 | 0.4 | C/A |
| NPR3 | rs1173743 | intron | 0.0943 | 0.368 | T/G |
| NPR3 | rs12522446 | intron | 1 | 0.112 | C/T |
| NPR3 | rs2270915 | exon | 0.9493 | 0.195 | A/G |
| NPR3 | rs3792758 | intron | 0.2911 | 0.225 | G/T |
| NPR3 | rs3811958 | intron | 0.6736 | 0.179 | T/C |
| NPR3 | rs976576 | intron | 0.3755 | 0.291 | A/G |

HWpval: Hardy-Weinberg p value, MAF: minor allele frequency,

Maj/Min: Major/Minor allele.

Table S1. SNP characteristics. Continued

| **Gene** | **SNP** | **Region** | **HWpval** | **MAF** | **Maj/Min** |
| --- | --- | --- | --- | --- | --- |
| NR3C2 | rs879206 | intron | 0.1572 | 0.133 | C/A |
| NR3C2 | rs907618 | intron | 0.9485 | 0.118 | T/C |
| NR3C2 | rs9307847 | intron | 0.4092 | 0.167 | T/C |
| NR3C2 | rs992556 | intron | 0.6538 | 0.267 | A/G |
| NR3C2 | rs10434100 | intron | 0.8563 | 0.148 | C/T |
| NR3C2 | rs10519951 | intron | 0.4041 | 0.147 | G/A |
| NR3C2 | rs10519959 | intron | 0.7361 | 0.11 | C/T |
| NR3C2 | rs11099681 | intron | 0.4583 | 0.292 | T/C |
| NR3C2 | rs11099690 | intron | 0.0321 | 0.178 | C/A |
| NR3C2 | rs13150372 | intron | 0.8279 | 0.451 | A/G |
| NR3C2 | rs1490453 | intron | 0.016 | 0.059 | C/T |
| NR3C2 | rs1512342 | intron | 0.1594 | 0.432 | T/C |
| NR3C2 | rs1512343 | intron | 0.075 | 0.114 | C/T |
| NR3C2 | rs1512344 | intron | 0.7251 | 0.244 | C/T |
| NR3C2 | rs1546529 | intron | 0.2769 | 0.333 | G/A |
| NR3C2 | rs1879829 | intron | 0.5211 | 0.331 | C/T |
| NR3C2 | rs1994624 | intron | 0.4771 | 0.263 | C/T |
| NR3C2 | rs2070951 | utr | 0.1059 | 0.256 | G/C |
| NR3C2 | rs2272089 | intron | 0.6185 | 0.239 | A/G |
| NR3C2 | rs2883930 | intron | 0.7565 | 0.178 | G/C |
| NR3C2 | rs3846317 | intron | 0.8633 | 0.351 | C/T |
| NR3C2 | rs3846320 | intron | 0.1024 | 0.166 | G/A |
| NR3C2 | rs3910044 | intron | 0.3149 | 0.474 | T/C |
| NR3C2 | rs3910054 | intron | 0.8685 | 0.262 | C/T |
| NR3C2 | rs4087963 | intron | 0.0066 | 0.477 | G/A |
| NR3C2 | rs4835136 | intron | 0.5208 | 0.11 | C/T |
| NR3C2 | rs4835490 | intron | 0.2626 | 0.083 | G/A |
| NR3C2 | rs4835493 | intron | 0.7022 | 0.291 | C/T |
| NR3C2 | rs5522 | exon | 1 | 0.148 | A/G |
| NR3C2 | rs5525 | exon | 0.7041 | 0.162 | G/A |
| NR3C2 | rs5534 | utr | 0.7226 | 0.204 | G/A |
| NR3C2 | rs6535578 | intron | 0.345 | 0.215 | A/C |
| NR3C2 | rs6535598 | intron | 0.3192 | 0.418 | T/G |
| NR3C2 | rs6810951 | intron | 0.5575 | 0.465 | G/T |
| NR3C2 | rs6855341 | intron | 0.0675 | 0.196 | C/T |
| NR3C2 | rs6857487 | intron | 1 | 0.383 | A/T |
| NR3C2 | rs7661835 | intron | 0.9293 | 0.134 | G/A |
| NR3C2 | rs7665528 | intron | 0.9567 | 0.324 | G/A |
| NR3C2 | rs7680420 | intron | 0.0139 | 0.182 | T/C |
| NR3C2 | rs7686433 | intron | 0.0045 | 0.104 | G/A |

HWpval: Hardy-Weinberg p value, MAF: minor allele frequency,

Maj/Min: Major/Minor allele.

Table S1. SNP characteristics. Continued

| **Gene** | **SNP** | **Region** | **HWpval** | **MAF** | **Maj/Min** |
| --- | --- | --- | --- | --- | --- |
| REN | rs10900555 | intron | 0.872 | 0.475 | T/C |
| REN | rs12750834 | intergenic | 0.6825 | 0.331 | G/A |
| REN | rs1464816 | intron | 0.9898 | 0.245 | G/T |
| REN | rs2368564 | intron | 0.8498 | 0.212 | C/T |
| REN | rs3795575 | intron | 0.5039 | 0.088 | C/T |
| REN | rs5705 | exon | 0.3125 | 0.101 | A/C |
| SCNN1B | rs1004749 | utr | 0.1492 | 0.341 | A/C |
| SCNN1B | rs168748 | intron | 0.8459 | 0.457 | G/A |
| SCNN1B | rs181835 | intergenic | 0.8926 | 0.329 | A/G |
| SCNN1B | rs2303153 | intron | 0.8828 | 0.121 | G/C |
| SCNN1B | rs238551 | intron | 0.7838 | 0.495 | A/G |
| SCNN1B | rs239349 | utr | 0.1561 | 0.477 | G/A |
| SCNN1B | rs250567 | intergenic | 0.0557 | 0.102 | G/A |
| SCNN1B | rs7205273 | intron | 0.0811 | 0.263 | C/T |
| SCNN1G | rs4247210 | intron | 0.8074 | 0.153 | G/C |
| SCNN1G | rs4299163 | intron | 0.8433 | 0.103 | G/C |
| SCNN1G | rs4401050 | intron | 0.0205 | 0.108 | C/T |
| SCNN1G | rs5723 | exon | 0.2751 | 0.06 | C/G |
| SCNN1G | rs5728 | utr | 0.9407 | 0.212 | A/G |
| SCNN1G | rs5735 | exon | 0.1407 | 0.172 | T/C |
| SCNN1G | rs9930846 | intergenic | 0.5534 | 0.06 | T/C |
| SELE | rs3917406 | intron | 0.8446 | 0.477 | C/T |
| SELE | rs3917419 | intron | 0.9921 | 0.15 | C/T |
| SELE | rs4786 | utr | 0.5496 | 0.44 | G/A |
| SELE | rs5368 | exon | 0.6306 | 0.276 | C/T |
| SELE | rs932307 | intron | 0.4286 | 0.48 | A/G |

HWpval: Hardy-Weinberg p value, MAF: minor allele frequency,

Maj/Min: Major/Minor allele.
